# Polymorphism of Alpha-Synuclein Amyloid Fibrils Depends on Ionic Strength and Protein Concentration

**DOI:** 10.3390/ijms222212382

**Published:** 2021-11-17

**Authors:** Mantas Ziaunys, Andrius Sakalauskas, Kamile Mikalauskaite, Vytautas Smirnovas

**Affiliations:** Institute of Biotechnology, Life Sciences Centre, Vilnius University, 10257 Vilnius, Lithuania; andrius.sakalauskas@gmc.vu.lt (A.S.); kamile.mikalauskaite@gmc.vu.lt (K.M.); vytautas.smirnovas@bti.vu.lt (V.S.)

**Keywords:** alpha-synuclein, amyloid, aggregation, ionic strength, polymorphism

## Abstract

Protein aggregate formation is linked with multiple amyloidoses, including Alzheimer‘s and Parkinson‘s diseases. Currently, the understanding of such fibrillar structure formation and propagation is still not sufficient, the outcome of which is a lack of potent, anti-amyloid drugs. The environmental conditions used during in vitro protein aggregation assays play an important role in determining both the aggregation kinetic parameters, as well as resulting fibril structure. In the case of alpha-synuclein, ionic strength has been shown as a crucial factor in its amyloid aggregation. In this work, we examine a large sample size of alpha-synuclein aggregation reactions under thirty different ionic strength and protein concentration combinations and determine the resulting fibril structural variations using their dye-binding properties, secondary structure and morphology. We show that both ionic strength and protein concentration determine the structural variability of alpha-synuclein amyloid fibrils and that sometimes even identical conditions can result in up to four distinct types of aggregates.

## 1. Introduction

Protein amyloid aggregation into insoluble fibrillar aggregates is linked with the onset and progression of multiple amyloidoses [1], including the widespread neurodegenerative Alzheimer‘s and Parkinson‘s diseases [2,3]. Despite years of research and growing number of such disorders [4], the overall process of protein aggregate formation is still not fully understood [5]. The complex nature of fibrillization and the resulting types of aggregates has led to the development of very few anti-amyloid drugs [6,7], with most potential compounds failing at various stages of clinical trials [8,9]. Since the number of patients affected by such protein aggregates is estimated to continue to rise over the next few decades [10,11], it is critically important to obtain a better understanding of protein fibrillization in the hopes of finding a potent cure or treatment.

Currently, it is known that multiple environmental factors can alter both the rate and mechanism of amyloid fibril formation. These include: temperature [12,13,14], agitation [15], protein concentration [16], ionic strength [17,18], denaturant concentration [19], pH [20], liquid-surface interfaces [21] and macromolecular crowding [22]. They affect primary nucleation, elongation, secondary processes (surface-mediated nucleation and fragmentation), fibril length and stability, as well as the structure of final aggregates [23]. Changes in solution pH value or ionic strength have been reported to affect fibril secondary structure/stability [24,25,26] and their interactions with amyloid-specific compounds, such as thioflavin-T, a fluorescent amyloid probe [26], or epigallocatechin-3-gallate, an aggregation inhibitor [27].

One of the most intensely studied amyloidogenic proteins is the Parkinson‘s disease-related alpha-synuclein (α-syn) [28]. During in vitro aggregation, it has been observed on multiple occasions that different ionic strength conditions affect the rate of aggregation and can lead to the formation of distinct types of α-syn fibrils [17,18,29,30]. It has also been reported that α-syn aggregates can undergo slight structural rearrangements when they are resuspended into different ionic strength solutions [26]. Salt concentration also altered their capacity to bind ThT [26] and different fibril types have even been shown to possess distinct ThT-binding properties [31]. In addition, it has recently been observed that small variations in the solution‘s pH value can significantly alter the effectiveness of anti-amyloid compounds [27,32]. Considering all of these factors, it seems that α-syn amyloid aggregation and the resulting structures are highly susceptible to the environmental conditions, where even a small shift in certain parameters can have a major influence.

Since spontaneous α-syn aggregation is known to be a highly stochastic process [33], there exists a possibility of different types of fibrils forming under the same conditions, as was shown for prion proteins [34]. There may also not be a clear-cut line in the structural transition reported to occur between lower and higher reaction solution ionic strength. For this reason, we tracked the aggregation kinetics of α-syn under six different ionic strength and five different protein concentration conditions, using large sample sizes in each case. The fibril ThT-binding/fluorescence parameters were used as an initial means of identifying sample variations [34,35], which were then examined using Fourier-transform infrared spectroscopy and atomic force microscopy. We show a shift in fibril structure variability based on the initial ionic strength and protein concentration, as well as outlier samples, which contain distinct structural and morphological features.

## 2. Results

**Figure 1:**
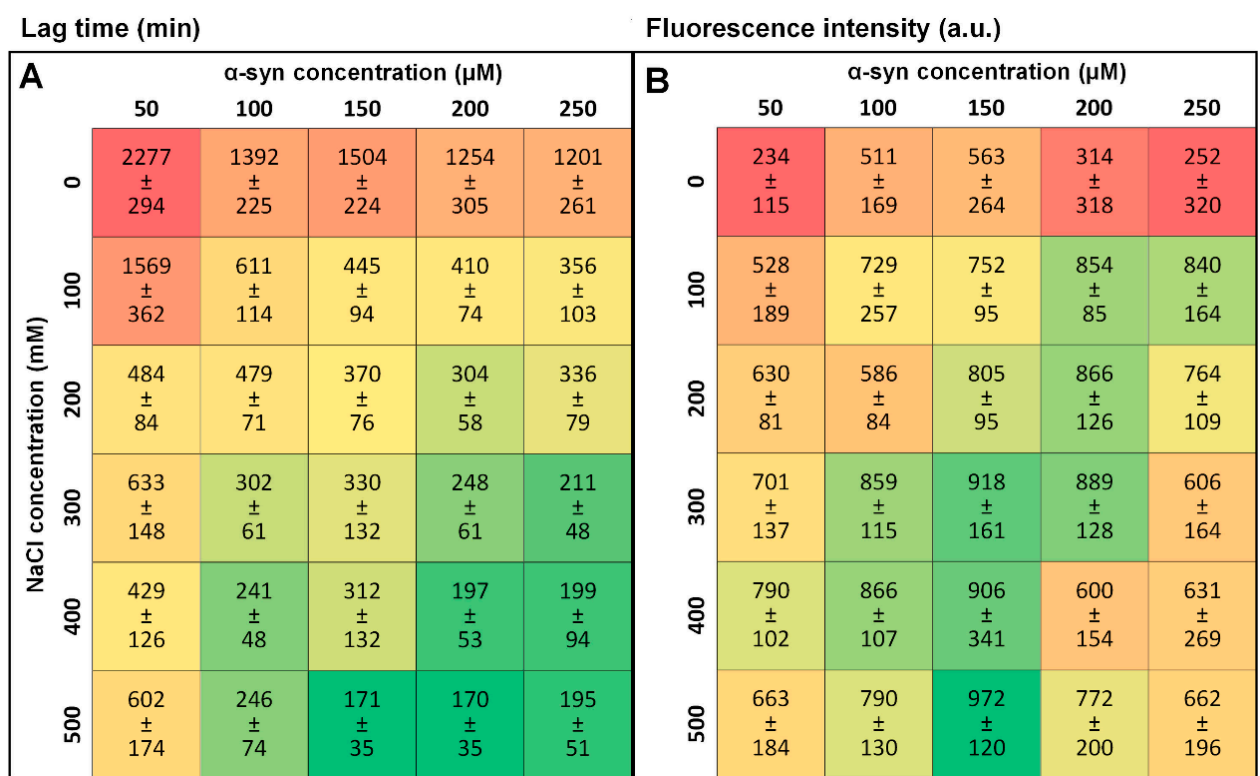
Alpha-synuclein aggregation lag time (**A**) and bound-ThT fluorescence intensity (**B**) dependence on protein concentration and solution ionic strength. Lag time and fluorescence intensity values and standard deviations were calculated from 90–96 repeats. Lag time color-coded boxes indicate a shift from long (red) to short lag times (green). Fluorescence intensity color-coded boxes indicate a shift from low (red) to high (green) intensity. Aggregation kinetic raw data are available as Supplementary Material.

Alpha-synuclein aggregation was tracked under six different ionic strength conditions (0–500 mM NaCl), using five different protein concentrations (50–250 µM), resulting in a total of thirty conditions. In each case, a full 96-well plate of identical reaction mixtures was examined in order to obtain a considerable distribution of kinetic data and fibril samples. The lag time values (Figure 1A) have an expected dependence on both protein and NaCl concentration, where an increase in either parameter reduces the lag time. From a general overview of this data, three observations can be made. First, there is a massive shift in lag time values when going from 0 mM to 100 mM NaCl. Under most protein concentrations, this change is 2- to 4-fold and this tendency does not persist throughout higher NaCl concentrations, where much less significant reductions are seen. In the case of 50 µM α-syn, the 3-fold change occurs between 100 mM and 200 mM NaCl, as opposed to 0–100 mM NaCl, indicating that such a shift depends on both the protein and salt concentration. Secondly, we see that under most conditions, the standard deviation value is quite large, especially taking into consideration the sizable number of repeats. This illustrates the highly stochastic nature of spontaneous α-syn aggregation. Finally, the lag time appears to reach a saturation at higher α-syn concentrations (150–250 µM), where an increase in protein concentration has minimal effect on the time needed for primary nuclei to form.

**Figure A1:**
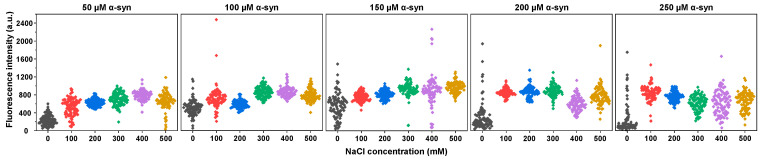
α-syn sample bound-ThT fluorescence intensity distribution at different ionic strength and protein concentration conditions.

After the aggregation reaction, all samples were cooled down to 25 °C and resuspended into equal ionic strength (500 mM NaCl) solutions, with equal protein concentrations (10 µM). After this, each sample‘s bound-ThT excitation-emission matrices (EEM) were scanned. Comparing the average fluorescence intensity of each set, we observe that the 0 mM NaCl sample intensity values are the lowest. They also have an extreme variation in intensity, as in some of the cases (Figure 1B, 200 µM, 250 µM), the standard deviation values are larger than the average value, which indicates a far-from-normal distribution (Figure A1). Under some conditions, there are even small subgroups of samples with either considerably smaller (50 µM α-syn, 500 mM NaCl) or larger (200 µM α-syn, 0 mM NaCl) fluorescence intensity values than the average value of their respective set (Figure A1). They also do not follow the same trend as lag time, with the highest average fluorescence values changing from lower ionic strength and high protein concentration to higher ionic strength and intermediate protein concentration (Figure 1B, green color-coded boxes).

**Figure 2:**
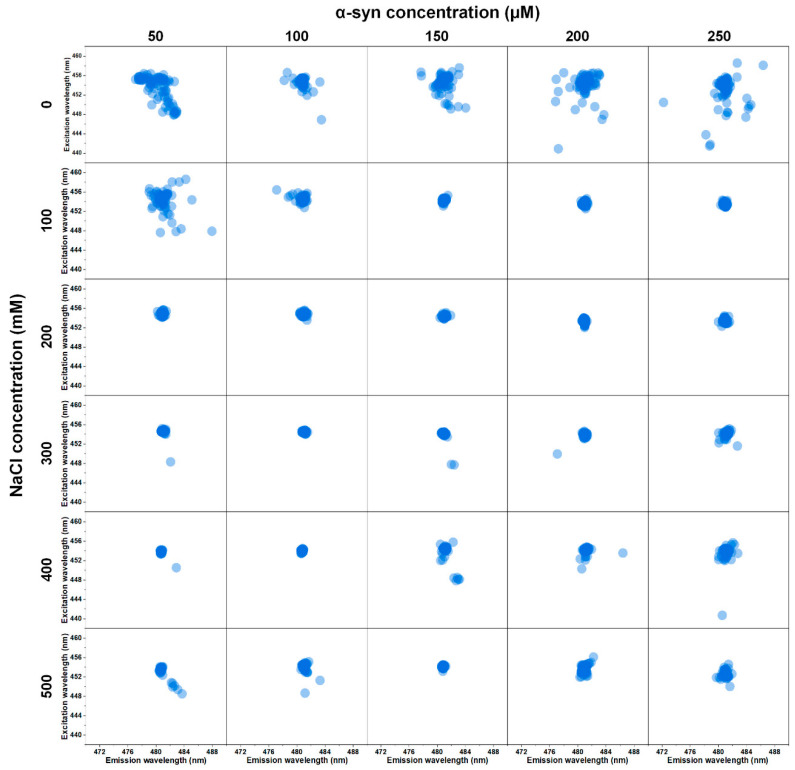
Excitation-emission matrix (EEM) maximum intensity positions of α-syn fibril-bound-ThT, determined for sample sets under thirty different environmental condition. Each position was calculated as described in the Materials and Methods section, at 25 °C, under identical ionic strength (500 mM NaCl) and protein concentration (10 µM) in order to have identical ThT binding conditions. EEM raw data are available as Supplementary Material.

Since there is a massive variance in bound-ThT fluorescence intensity and it is known that different α-syn fibrils can possess specific ThT-binding properties [31], each EEM‘s “center of mass“ was calculated, in order to determine the maximum excitation and emission wavelengths. An overview of all the EEM matrices displays three distinct regions of variability (Figure 2). At low ionic strength conditions, the EEM maximum positions are spread out over a large area, with multiple outliers in each case (samples that do not belong to the main cluster). At intermediate ionic strength conditions, the variability is greatly reduced, with some sample sets having only a single EEM maximum position. Going further, higher ionic strength and protein concentrations increase the variability, but not to the same extent as low ionic strength conditions. Taking all of this into consideration, it appears that both low ionic strength and high ionic strength and protein concentrations lead to the formation of fibrils with distinct ThT-binding properties, while intermediate samples all seem to have one dominant type of binding.

Considering that each sample set has different bound-ThT intensity values and many sets have one or more outliers, the samples were divided into two groups. For the first, one sample was chosen from each condition, which had an average fluorescence intensity and belonged to the main EEM position cluster. For the second group, the lowest and highest fluorescence samples (fringe samples), as well as one or more outliers (very distinct EEM position) from each condition were selected. The aim of both groups was to determine the most common type of fibrils to occur at every given condition and also to analyze fringe samples and outliers.

**Figure 3:**
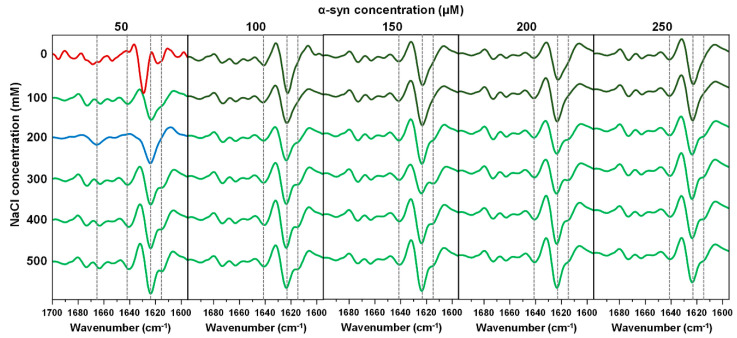
Second derivative FTIR spectra of α-syn fibril samples, which have an average fluorescence intensity and an EEM position in the main cluster. Samples from all thirty conditions were replicated in their respective initial reaction solutions in order to obtain higher quantity of fibrils for a better quality FTIR spectra and to ensure their replication. Spectra which contain significant similarities are color-coded identically. FTIR raw data are available as Supplementary Material.

The selected fibrils were replicated under their respective initial reaction conditions, in order to both increase the mass of aggregates for examination by FTIR, as well as to ensure that these structures are capable of self-replication. The FTIR spectra second derivatives of all average samples (first group) reveal that under many conditions, there are two dominant secondary structures (Figure 3). At higher ionic strength conditions, the most dominant type of fibril (Figure 3, light green) displays a main minimum at 1624 cm^−1^ in second derivative of FTIR spectra, which is related to beta-sheet hydrogen bonds [36]. The less pronounced band at 1615 cm^−1^ can be associated with stronger hydrogen bonds in the beta-sheet structure. There are also minima at 1641 cm^−1^ (weak hydrogen bonds), as well as 1663 cm^−1^ and 1673 cm^−1^ (turn/loop motifs). The other dominant structure appears at lower ionic strength conditions (Figure 3, dark green), whose second derivative FTIR spectrum is similar to the aforementioned fibrils, but contains only a minor shoulder at 1615 cm^−1^, which indicates a lower amount of stronger hydrogen bonds in the beta-sheet structure.

Interestingly, there are two distinct types of fibrils at lower protein concentrations and ionic strength. In the case of 200 mM NaCl and 50 µM α-syn, we observe a second derivative FTIR spectrum, which has a main minimum at a similar position to both dominant fibril types, but which lacks stronger (1615 cm^−1^) and weaker (1641 cm^−1^) hydrogen bonds, and which only contains one minimum in the turn/loop motif region (1665 cm^−1^). In the case of 0 mM NaCl and 50 µM α-syn, the second derivative spectrum contains a main minimum at 1629 cm^−1^, which is related to weaker hydrogen bonds in the beta-sheet structure and a less expressed minimum at 1618 cm^−1^ (related to stronger hydrogen bonds), making it highly distinct from the rest.

**Figure 4:**
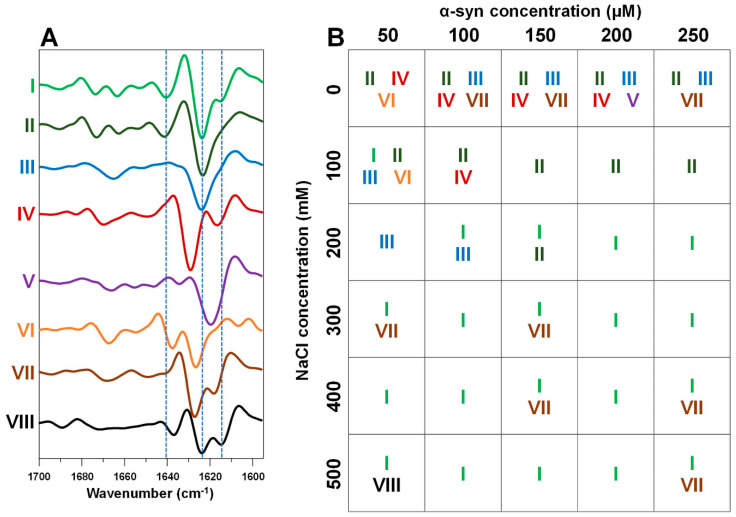
Second derivative FTIR spectra of fringe and outlier α-syn fibril samples (**A**). Samples from all thirty conditions were replicated in their respective initial reaction solutions in order to obtain higher quantity of fibrils for a better quality FTIR spectra and to ensure their replication. Conditions where these spectra were observed (**B**), with each spectrum assigned with a color-coded Roman numeral. FTIR raw data are available as Supplementary Material.

Seeing as there were four distinct secondary structure aggregates present in the set containing the most likely-to-occur samples, the fringe and outlier fibrils were also examined and compared. In this case, four additional second derivative FTIR spectra were discovered (Figure 4 V–VIII), which had different features from the initial four spectra (Figure 3 and Figure 4 I–IV). One spectrum, found among 0 mM NaCl samples (Figure 4 V), had one main minimum at 1620 cm^−1^ and very few other features, indicating a dominant presence of one type of hydrogen bonds in the beta-sheet structure. Another spectrum, found among low ionic strength and protein concentration (Figure 4 VI) had a main minimum at 1623 cm^−1^ and another minimum at 1637 cm^−1^, which suggests two types of hydrogen bonding, which are dissimilar to other fibrils. The next spectrum, which was observed in many instances among outlier samples (Figure 4 VII), shared some similarities to the IV spectrum; however, the main minimum was shifted towards lower wavenumbers, indicating stronger hydrogen bonds. The position at 1619 cm^−1^ was also more of a shoulder, rather than a separate minimum. Finally, the outlier, which only appeared at 500 mM NaCl and 50 µM α-syn, had similar minima to the dominant type fibril spectra (1615 and 1641 cm^−1^), but had a shifted minima at 1636 cm^−1^, as well as no clearly discernible turn/loop motifs.

Based on the distribution of these eight spectra (Figure 4B), it is quite clear that the most variability is present at low ionic strength and protein concentration, with 3–4 distinct conformations present in most cases. This variance in fibril secondary structures correlates with the EEM position variability, further solidifying the fact that low ionic strength and protein concentrations lead to not only distinct dominant type of fibril, but also to an abundance of different aggregates.

**Figure 5:**
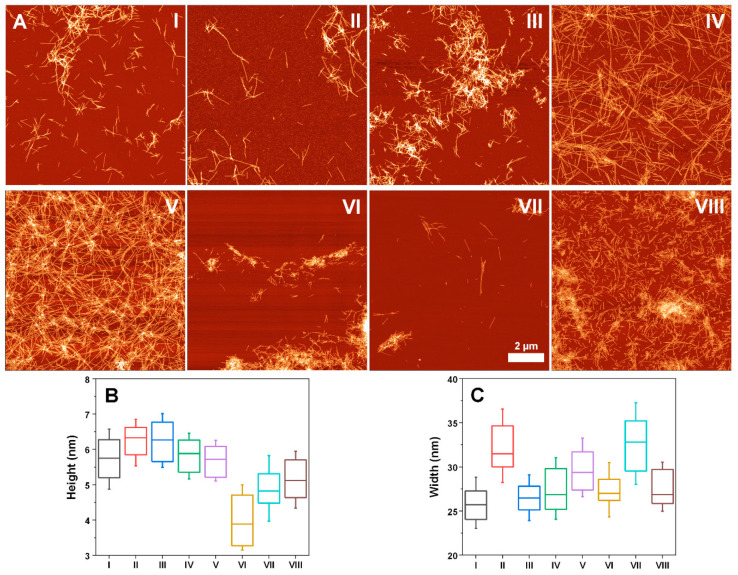
Atomic force microscopy images of α-syn fibrils, which had distinct second derivative FTIR spectra (**A**) and their height (**B**) and width (**C**) distribution (*n* = 50). Roman numerals correlate with Figure 4 FTIR spectra. Box plots indicate the interquartile range and the error bars are one standard deviation.

The eight different fibril types were further examined using AFM (Figure 5). Based on a simple visual inspection, the I, II and III samples appear to be quite similar; however, the II sample has significantly wider fibrils (32 nm, as opposed to 26–27 nm). Both IV and V fibrils form long, intertwined networks, which are not observed in any of the other samples and both their height and width are within the margin of error. The VI sample contains mostly aggregate clusters and the fibril height is the lowest out of all eight samples. The VII sample likely contains similar large clusters, as very few non-bound fibrils could be observed. They did, however, have a lower height when compared to most other aggregates, as well as a width similar to the II sample. Finally, the VIII sample had relatively short fibers, which form similar networks and clusters as IV and V fibrils, their height is also similar to the VII sample, while their width does not pertain any notable deviations.

To examine if different morphologies and secondary structures have an effect on fibril self-replication potential, the eight different aggregate types were resuspended into identical ionic strength (500 mM NaCl) and non-aggregated α-syn (50 µM) solutions. Their fibrillization was tracked as described in the Materials and Methods section. Comparing all fibril types, it appears that type IV and VII aggregates lead to the most significant reduction in lag time values (Figure A3A). Oppositely, III, V and VI fibrils have the least seeding potential, with the remaining types causing an intermediate reduction in lag time. The V and VI aggregates also have the lowest elongation rates (Figure A3B), while all other types have significantly higher values. Based on these observations, it appears that there is no clear correlation between seeding capacity and fibril morphology, secondary structure or initial preparation conditions.

## 3. Discussion

Based on these data and previous reports, it is quite clear that both ionic strength and protein concentration play a significant role in determining the type of α-syn fibrils during spontaneous aggregation. In order to evaluate the effect of each factor, we have to discuss the correlation between all the present data, including aggregation kinetics, variation in conformations and aggregate morphology.

First, we can see that all protein concentrations at 0 mM NaCl and 50 µM α-syn at 100 mM NaCl experience significantly longer lag times, when compared to all other conditions. Such long time-frames could potentially allow for the formation of distinct nuclei, which, in turn, lead to structurally and morphologically different fibrils. These aforementioned conditions coincide with both the large variability of EEM maximum positions (Figure 2), secondary structure (Figure 4) and fibril morphology (Figure 5). However, the long aggregation times do not explain why there is a shift in the dominant type of aggregate, which occurs between 100 mM and 200 mM NaCl, rather than between 0 mM and 100 mM. In some cases, aggregation at 100 mM occurs quicker or similarly to 200 mM, but the resulting aggregates still pertain their ionic strength-specific secondary structure. Coincidentally, this transition in dominant fibril type occurs at an ionic strength similar to PBS, which is another buffer solution often used in alpha-synuclein studies and which also leads to the formation of a mixture of fibrils [37]. This means that ionic strength plays a crucial role during nuclei formation, where the presence of sodium and chloride ions alter the type of nucleus that forms, likely by influencing protein electrostatic interactions.

The concentration of α-syn also appears to play a role in determining the type of fibril, especially visible at lower ionic strength conditions. This factor, however, does not seem to have a significant effect at high protein and ionic strength conditions, where both the aggregation kinetics, as well as types of fibrils experience lower variation. This could be due to the reaction reaching the highest rate of nucleation and elongation, where the dominant type of fibril becomes the one that forms and elongates the quickest. This would leave no time for other nuclei formation and essentially limit the conformational variations.

One especially interesting and unexpected aspect of this work was the discovery of eight distinct fibril types, which have specific secondary structure motifs and, in some cases, morphologies. Based on previously reported α-syn aggregation experiments [18,29], one would expect to only observe a limited fibril variation, especially under similar conditions. On the one hand, it is quite interesting that α-syn is capable of forming up to four distinct types of fibrils under identical conditions (mostly seen at low ionic strength). This is a far greater variation than we have demonstrated in the case of prion proteins, which were capable of forming two different types of fibrils under identical conditions [34]. On the other hand, this means that during experimental procedures, α-syn can aggregate into more than one type of fibril. Taking into consideration the different secondary structures and morphologies, this random variation could significantly alter the outcome of any potential drug molecule assay or aggregation kinetic experiment. Another factor which may also complicate data interpretation is the vast dispersion of sample fluorescence intensities. Apart from distinct fibrils having significantly lower or higher bound-ThT fluorescence intensity, there were also substantial differences in intensity between fibrils that had similar secondary structures. This means that the assumption of ThT fluorescence relating to the concentration of fibers would be incorrect in the case of α-syn aggregation experiments.

The appearance of multiple distinct conformation α-syn fibrils under identical conditions may also be one of the steps in the complex mechanism of pathogenesis in Parkinson’s disease and other synucleinopathies. Currently, the exact mode of alpha-synuclein-related disorder onset and propagation is not fully known, with multiple possible mechanisms proposed, such as prion-like spreading [38] and trans-synaptic α-syn propagation [39,40]. It has also recently been shown by Ferreira et al. [41] and Peng et al. [42] that certain α-syn fibril types have a considerably higher neurodegenerative potential than others. Combined with the findings in this work, it is possible that the polymorphism of α-syn fibrils at physiological ionic strength/protein concentration results in some of the aggregates having a significantly higher self-replication potential (Figure A3) and possible higher cytotoxicity.

As a positive note, the thirty different environmental conditions revealed certain sets of ionic strength and protein concentrations, which experienced minimal variation in the type of fibril that forms. As an example, the 200 mM NaCl and 200 µM α-syn conditions resulted in samples, which had identical EEM maximum positions, as well as a single type of secondary structure and morphology. Such conditions, which lead to homogenous fibrillization, can be used to avoid conformational variations during assays which employ α-syn aggregation and aid in amyloid research. Taking everything into consideration, spontaneous alpha-synuclein aggregation is a stochastic process, whose results depend highly on both the solution’s ionic strength, as well as protein concentration. Conformational variation of the resulting fibrils depends on both parameters, with certain conditions leading to the formation of up to four structurally and morphologically distinct fibrils, while others result in all samples containing a single type of aggregate.

## 4. Materials and Methods

### 4.1. Alpha-Synuclein Aggregation

Alpha-synuclein was purified as described previously [43], lyophilized and stored at −20 °C prior to use. Before each aggregation reaction, α-syn powder was dissolved in a 20 mM potassium phosphate buffer (pH 7.4), containing 0–500 mM NaCl (further referred to as the reaction buffer) and filtered through a 0.22 µm syringe filter. The protein concentration was determined by scanning sample absorbance at 280 nm using a Shimadzu (Kyoto, Japan) UV spectrophotometer (ε_280_ = 5960 M^−1^cm^−1^). The resulting solutions were then combined with their respective reaction buffers and a 10 mM thioflavin-T (ThT) stock solution to yield samples containing 50–250 µM final protein concentration and 100 µM ThT (10 mL total volume). The solutions were then distributed to 96-well non-binding plates, with each well containing 100 µL protein solution, a 3 mm glass-bead and sealed using Nunc sealing tape. The plates were then placed in a Clariostar Plus plate reader and incubated at 37 °C under constant 600 RPM orbital agitation. The presence of a 3 mm glass-bead and constant agitation minimized the possibility of alpha-synuclein liquid-liquid phase separation [44]. Measurements were taken every 5 min using excitation and emission wavelengths of 440 nm and 480 nm, respectively. After aggregation, the plates were stored at 4 °C.

For seeded aggregation, fibrils were centrifuged at 12,000 RPM for 15 min, after which the supernatant was removed and the fibril pellets were resuspended into a 20 mM potassium phosphate buffer (pH 7.4), containing 500 mM NaCl. The fibril solutions were then combined with a 100 µM α-syn solution (500 mM NaCl), ThT stock solution (10 mM) and 20 mM potassium phosphate buffer (pH 7.4), containing 500 mM NaCl to final solutions containing 50 µM α-syn, 2.5 µM fibrils (fibril concentration is based on aggregated monomer concentration, assuming 100% fibrillization) and 100 µM ThT. Solution aggregation was tracked as described in the non-seeded aggregation section.

**Figure A2:**
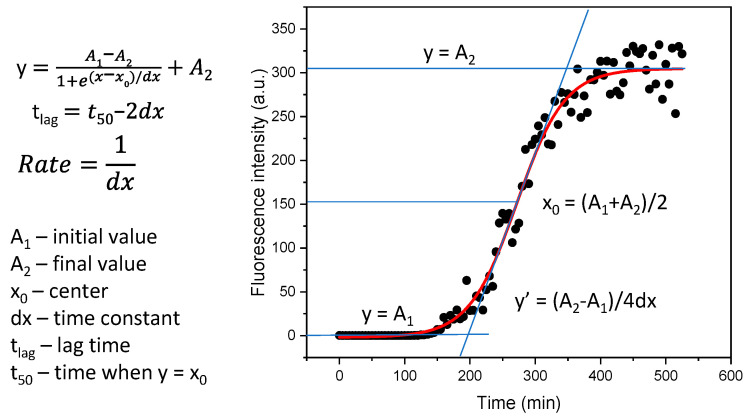
Boltzmann sigmoidal equation fit example with lag time (t_lag_) and elongation rate calculation.

**Figure A3:**
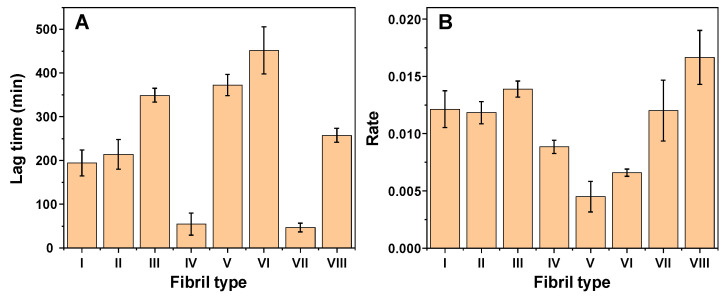
Lag time (**A**) and elongation rate (**B**) of all eight fibril types under identical ionic strength (500 mM NaCl), non-aggregated α-syn (50 µM) and initial fibril concentration (2.5 µM). Lag time and elongation rate values were calculated as shown in Figure A2.

Aggregation kinetic data were analyzed using Origin 2018 software (OriginLab Corporation, Northampton, MA, USA). Each kinetic curve was fit using a sigmoidal Boltzmann equation. The lag time was determined as shown in the Figure A2. In a rare set of cases, the aggregation curve data quality was not sufficient for analysis. In these instances, the samples were taken out of the batch.

### 4.2. Excitation-Emission Matrices

An aliquot of each sample was removed from the 96-well plates and diluted with 20 mM potassium phosphate buffers (pH 7.4), containing either 500 mM or 650 mM NaCl and 100 µM ThT, in order to make every sample contain 500 mM NaCl and 10 µM protein. An identical protein and NaCl concentration was required to compare ThT binding/fluorescence parameters, as both factors affect the concentration of bound ThT [26]. The addition of fresh ThT minimized the loss of the dye molecule due to hydroxylation during the aggregation experiment [45]. After the samples were prepared, they were placed into 96-well plates, and their bound-ThT excitation-emission matrices were scanned and generated using Clariostar Plus platereader and Clariostar MARS software. Emission intensity in the range from 470 nm to 490 nm was scanned using a set excitation wavelength of 450 nm. Afterwards, emission intensity at 480 nm was scanned using an excitation range from 440 nm to 460 nm. In each case, a 1 nm step size was used. The resulting data were combined into an EEM using Clariostar MARS software (3D spectrum function). The EEM “center of mass” was determined as described previously [46].

### 4.3. Fourier-Transform Infrared Spectroscopy (FTIR)

In order to prepare samples for FTIR, the selected fibril samples were first replicated by combining them with their respective reaction solutions (same NaCl and protein concentration as the initial samples) in a 1:4 ratio (20% fibril seed, assuming a complete fibrillization of the initial samples) and incubating them as described in the initial aggregate preparation section. The resulting samples were then centrifuged at 12,000 RPM for 15 min. The supernatant was removed and the fibril pellets were resuspended into 500 µL of D_2_O, containing 500 mM NaCl (the addition of NaCl improved fibril sedimentation and created an equal ionic strength environment for all samples). The centrifugation and resuspension procedure was repeated four times. Finally, the fibril pellets were resuspended into 100 µL of D_2_O, containing 500 mM NaCl and mixed vigorously for 10 s. For each sample, 256 interferograms of 2 cm^−1^ resolution were scanned using a Bruker (Billerica, MA, USA) Invenio S FTIR spectrometer (equipped with MCT detector) at room temperature. D_2_O and water vapor noise spectra were subtracted from each sample spectrum, which was then baseline corrected (1700–1595 cm^−1^) and normalized to the area of Amide I‘ band. All data processing was done using GRAMS software.

### 4.4. Atomic Force Microscopy

Before depositing the fibril samples, the mica surface was modified with (3-aminopropyl)triethoxysilane (APTES). Then, 1% (% v.v) APTES solution (30 µL) was spread on the surface of the mica, incubated at room temperature for 5 min, gently washed with 2 mL of H_2_O and dried using airflow. For AFM measurements, 30 µL aliquots of samples used in the EEM measurements were taken (identical protein and NaCl concentration) and placed on APTES-modified mica and left to adsorb for 60 s. The mica were then gently washed with 3 mL of H_2_O and dried using airflow. AFM measurements were done as described previously [27]. In short, high-resolution AFM images were acquired using Dimension Icon (Bruker, Billerica, MA, USA)) atomic force microscope, operating in tapping-mode. The images were flattened and analyzed using Gwyddion 2.5.5 software. Fibril cross-sectional height and width were determined from line profiles, perpendicular to the fibril axes (only separate and non-clumped fibrils were measured).

## Data Availability

The data presented in this study are available in Supplementary Material. It includes raw kinetic, EEM and FTIR data.

## Supplementary Materials

The following are available online at https://www.mdpi.com/article/10.3390/ijms222212382/s1.
